# Comprehensive characterization of circular RNAs in ~ 1000 human cancer cell lines

**DOI:** 10.1186/s13073-019-0663-5

**Published:** 2019-08-26

**Authors:** Hang Ruan, Yu Xiang, Junsuk Ko, Shengli Li, Ying Jing, Xiaoyu Zhu, Youqiong Ye, Zhao Zhang, Tingting Mills, Jing Feng, Chun-Jie Liu, Ji Jing, Jin Cao, Bingying Zhou, Li Wang, Yubin Zhou, Chunru Lin, An-Yuan Guo, Xi Chen, Lixia Diao, Wenbo Li, Zhiao Chen, Xianghuo He, Gordon B. Mills, Michael R. Blackburn, Leng Han

**Affiliations:** 1 Department of Biochemistry and Molecular Biology, McGovern Medical School at The University of Texas Health Science Center at Houston, Houston, TX 77030 USA; 2 School of Computer Science, Wuhan University, Wuhan, 430072 Hubei People’s Republic of China; 3 Department of Bioinformatics and Systems Biology, Hubei Bioinformatics & Molecular Imaging Key Laboratory, Key Laboratory of Molecular Biophysics of the Ministry of Education, College of Life Science and Technology, Huazhong University of Science and Technology, Wuhan, 430074 Hubei People’s Republic of China; 4 Center for Translational Cancer Research, Institute of Biosciences and Technology, Texas A&M University, Houston, TX 77030 USA; 5 Department of Molecular and Cellular Biology, Baylor College of Medicine, Houston, TX 77030 USA; 6 State Key Laboratory of Cardiovascular Disease, Fuwai Hospital, National Center for Cardiovascular Diseases, Chinese Academy of Medical Sciences and Peking Union Medical College, Beijing, 100037 People’s Republic of China; 7 Department of Molecular and Cellular Oncology, The University of Texas MD Anderson Cancer Center, Houston, TX 77030 USA; 8 Department of Bioinformatics and Computational Biology, The University of Texas MD Anderson Cancer Center, Houston, TX 77030 USA; 9 Fudan University Shanghai Cancer Center and Institutes of Biomedical Sciences, Shanghai Medical College, Fudan University, 270 Dong An Road, Shanghai, 200032 People’s Republic of China; 10 Knight Cancer Institute, Oregon Health and Science University, Portland, OR 97239 USA

## Abstract

**Background:**

Human cancer cell lines are fundamental models for cancer research and therapeutic strategy development. However, there is no characterization of circular RNAs (circRNAs) in a large number of cancer cell lines.

**Methods:**

Here, we apply four circRNA identification algorithms to heuristically characterize the expression landscape of circRNAs across ~ 1000 human cancer cell lines from CCLE polyA-enriched RNA-seq data. By using integrative analysis and experimental approaches, we explore the expression landscape, biogenesis, functional consequences, and drug response of circRNAs across different cancer lineages.

**Results:**

We revealed highly lineage-specific expression patterns of circRNAs, suggesting that circRNAs may be powerful diagnostic and/or prognostic markers in cancer treatment. We also identified key genes involved in circRNA biogenesis and confirmed that TGF-β signaling may promote biogenesis of circRNAs. Strikingly, we showed that clinically actionable genes are more likely to generate circRNAs, potentially due to the enrichment of RNA-binding protein (RBP) binding sites. Among these, circMYC can promote cell proliferation. We observed strong association between the expression of circRNAs and the response to drugs, especially those targeting chromatin histone acetylation. Finally, we developed a user-friendly data portal, CircRNAs in cancer cell lines (CircRiC, https://hanlab.uth.edu/cRic), to benefit the biomedical research community.

**Conclusions:**

Our study provides the characterization of circRNAs in cancer cell lines and explored the potential mechanism of circRNA biogenesis as well as its therapeutic implications. We also provide a data portal to facilitate the related biomedical researches.

**Electronic supplementary material:**

The online version of this article (10.1186/s13073-019-0663-5) contains supplementary material, which is available to authorized users.

## Background

CircRNA, a class of non-coding RNA characterized by a covalently closed circular structure [1], is emerging as a surprising, pervasive feature of gene expression with the discovery of its abundance among species [2, 3]. CircRNAs are generated by a “backsplicing” process in which a downstream 5′ splice site backsplices to an upstream 3′ splice site [4], and this process is regulated by both *cis* elements and *trans* protein factors [4]. For example, some RNA-binding proteins (RBPs), including QKI and MBL, can enhance circRNA formation via bridging flanking introns together [5, 6], while some RBPs (e.g., PTBP1) can reduce the formation of circRNAs [7, 8]. CircRNAs may also be regulated by specific biological processes, such as epithelial–mesenchymal transition (EMT) [6]. However, the detailed mechanism underlying circRNA biogenesis remains largely unknown.

Emerging evidence has shown important roles of circRNAs in human diseases, e.g., cancer [9, 10]. CircRNAs derived from oncogenic fusion genes, such as circMLL/AF9, can contribute to tumor-promoting properties [11], while circ-FBXW7, which is derived from a tumor-suppressive E3 ligase, can repress tumorigenesis [12]. Several databases have been developed for exploring the link between circRNAs and human diseases, such as the cancer-specific circRNA database (CSCD) [13]. Recent studies characterized the circRNAs, suggesting their functional roles to promote cell proliferation [14–16] and could serve as biomarkers in cancer [17, 18].

Human cancer cell lines are important experimental models and have facilitated fundamental discoveries in cancer research [19, 20]. Several large-scale drug sensitivity studies have been conducted in cancer cell lines to explore the diversity of therapeutic response [19, 21, 22]. These studies have systematically characterized multiple layers of genomic data in large numbers of cancer cell lines, including mutations, copy number variations, mRNA expression [19, 23], protein expression [21], alternative polyadenylation [22], and metabolism [24], coupled with pharmacological drug response profiles. These studies represent valuable resources to guide rational cancer therapeutic strategies. There is limited knowledge about the biogenesis, functional consequences, and therapeutic liability of circRNAs in cancer cell lines; to fill this gap, we systematically analyzed global circRNA expression in a large panel of cancer cell lines from the Cancer Cell Lines Encyclopedia (CCLE) [19] to enlarge the translational utility of cancer cell lines.

## Methods

### Data resources

We downloaded paired-end RNA-seq BAM files of 935 cancer cell lines from The NCI’s Genomic Data Commons (https://portal.gdc.cancer.gov/legacy-archive) [19] as previously described [25]. We downloaded the CCLE gene expression data from the Cancer Target Discovery and Development Network (https://ocg.cancer.gov/programs/ctd2/data-portal) [26], mutation data from CCLE data portal (https://portals.broadinstitute.org/ccle) [19], and reverse-phase protein array data from the MD Anderson Cell Lines Project (http://tcpaportal.org/mclp) [21]. Sequencing depths of 935 CCLE cancer cell line RNA-seq samples ranged from 3.4 M reads to 455.3 M reads with medium as 167.3 M reads. We downloaded drug sensitivity data and compound annotation information from three pharmacogenomic resources: Genomics of Drug Sensitivity in Cancer (GDSC, http://www.cancerrxgene.org/) [27], Cancer Therapeutics Response Portal (CTRP, https://portals.broadinstitute.org/ctrp/) [28], and CCLE (https://portals.broadinstitute.org/ccle) [19] as previously described [29, 30]. Gene expression *z*-scores of canonical EMT markers were obtained from CCLE database to calculate EMT score [31].

### Identification of circRNAs in cancer cell lines

To achieve the most reliable precision and sensitivity, as well as balanced performance [32], we combined four circRNA prediction methods, CIRI2 [33], CIRCexplorer2 [34], circRNA_finder [35], and find_circ [3], with default settings, to identify circRNAs from the CCLE RNA-seq dataset. We further required each circRNA to be detected by at least two methods, with backsplicing reads ≥ 2 in each cancer cell line. For each circRNA detected by at least two detection tools with at least 2 backsplicing reads, we calculated the average number of backsplicing reads across detectable tools. The average number of backsplicing reads was normalized by total number of reads in each cell line.

### Identification of lineage-specific mRNAs and lineage-specific circRNAs

To identify the lineage-specific mRNAs, we compared the median expression level $\mathrm{med}\left({e}_j^{(l)}\right)$ of a gene (*j*) in a particular cancer lineage (*l*) to the median and interquartile range of its expression across all samples, as described by Sonawane et al. [36]:

$$ {s}_j^{(l)}=\left(\mathrm{med}\left({e}_j^{(l)}\right)-\mathrm{med}\left({e}_j^{\left(\mathrm{all}\right)}\right)\right)/ IQR\left({e}_j^{\left(\mathrm{all}\right)}\right). $$

We defined a gene with specificity score ${s}_j^{(l)}>0.5$ as being specific to cancer lineage *l*. The cutoff 0.5 was determined dynamically under that approximately half of all genes are identified as tissue-specific in our dataset [36]. We defined the circRNAs that can be detected in only one cancer lineage as lineage-specific circRNAs.

### Analysis of the biogenesis of circRNAs

To examine the effect of genes on circRNA biogenesis, we assessed the correlation between gene expression and normalized backsplicing read numbers by normalizing to the total number of reads (library size) in each cancer cell line. We defined significant correlation between gene expression level and normalized backsplicing reads as the absolute value of Spearman’s correlation > 0.3 and false discovery rate (FDR) adjuested *P *value < 0.05.

To assess the gene expression signatures or pathway associated with circRNA biogenesis, we first classified the cell lines into two groups (circRNA-low and circRNA-high) based on the backsplicing read numbers of all circRNAs. We then used GSEA (http://software.broadinstitute.org/gsea/index.jsp) [37] to test whether any hallmark gene sets are significantly enriched in the circRNA-high group. We considered the gene signatures or pathways with FDR < 0.05 as significantly enriched.

### TGF-β treatment

Human lung carcinoma cells (A549) were purchased from the American Type Culture Collection. This cell was used until between passages 2 and 5. SIS3, the Smad3 inhibitor for phosphorylation on serine 423/425, was purchased from Tocris. TGF-β1 was purchased from GenDEPOT. A549 cells were pretreated with 5 μM of SIS3 for 1 h, followed by TGF-β1 treatment at 5 ng/mL in the presence or absence of SIS3 for 30 min. Collagen 1 was used as a positive maker for the TGF-β experiment as described in previous studies [38, 39]. The cells were lysed by RIPA buffer that contained protease inhibitors (Thermo Scientific). The concentration of proteins was measured by BCA assay and used for western blot analysis.

### RNA sequencing

Cells were treated with TGF-β1 and SIS3, and RNAs were extracted by using the Quick-RNA MiniPrep kit from ZYMO Research. rRNA depletion was performed in NEBNext Ultra II Directional RNA Library Prep Kit implemented by Novogene prior to RNA-seq. RNA-seq was performed on an Illumina HiSeq platform by Novogene. RNA-seq data was analyzed using our analytic pipeline for CCLE samples as described in the previous section. The RNA-seq data have been submitted to the NCBI Gene Expression Omnibus (GSE119145).

### Permutation test

We randomly selected the same number of genes as the number of clinically actionable genes (total number 135) from the list of background genes (human protein-coding genes with circRNA expressed in at least five samples, excluding clinically actionable genes, *n* = 3693), with similar gene length, exon length, intron length, exon count, or expression level (within the range of those of clinically actionable genes). For testing enrichment of generation of circRNA from clinically actionable genes, we examined the frequencies of the circRNAs detected in the randomly selected gene sets from background genes. For testing enrichment of RBP binding peaks in clinically actionable genes, we retrieved RBP binding sites from CLIP-seq datasets for 37 RBPs deposited in starBase (http://starbase.sysu.edu.cn/) [40]. We collected binding peaks and calculated the number of peaks for each clinically actionable gene and background protein-coding gene. We repeated this process 10,000 times, and based on the observed distributions of these permutations, we assessed the statistical significance of enrichment of circRNAs in clinically actionable genes relative to random expectation.

### PCR amplification of linear MYC and circMYC upon RNase R treatment

We used specific primers to PCR linear MYC [F 5′- TAGTGGAAAACCAGCAGCCT-3′ R 5′- AGAAATACGGCTGCACCGAG-3′] and circMYC [F 5′ CTCACAGCCCACTGGTCCTC-3′ and R 5′- TCCAGCAGAAGGTGATCCAG-3′]. RNAs were isolated from the MDA-MB-231 that were transfected with either circMYC or control plasmids. Two micrograms of RNAs was incubated with RNase R (VWR) for 3 h to degrade linear RNAs, followed by TRIzol™-chloroform-based RNA purification. cDNAs were generated from the RNAs and were used for PCR reactions. No reverse transcriptase (No RT) was used as a negative control and the circMYC was used as a positive control to localize the appearance of circMYC. The treatment with RNase R and without circMYC overexpression was send out for Sanger sequencing with the primer as 5′-CATCAGCACAACTACGCAGC-3′.

### Overexpression and knockdown of circMYC

cDNA of MYC was amplified via PCR from the human cDNA using the following primers: F 5′-GGTCAGAGTCTGGATCACCTTC-3′ and R 5′-ACTGTCCAACTTGACCCTCTTG-3′. After the PCR amplification and purification, the cDNA part that undergoes circularization was cloned into the pcDNA3.1 circRNA mini vector which induces circularization of mRNA via the Gibson cloning [41]. MDA-MB-231 cells were transfected with the control or circMYC plasmid for 6 h by the JetPRIME™ reagent from Polyplus-transfection®. After 6 h of the transfection, the cells were further cultured with high-glucose DMEM media (GenDEPOT).

The MDA-MB-231 cells were plated onto TTP culture dishes. The cells were transfected with control siRNA or siRNA specific targets the junction of circular MYC for 8 h (INTERFERin® from PolyPlus-transfection®). The siRNA sequence is 5′ ACAGUGUCAGAGUCUGGAUCACCTT 3′+ 5′ AAGGUGAUCCAGACUCUGACACUGUCC 3′−. One day after the siRNA transfection, the cells were further transfected with control or circMYC plasmids for 6 h (JetPRIME™ from PolyPlus-transfection®).

### Western blot, RT-qPCR, and WST-1 cell proliferation

After 1 day with overexpression of circMYC, the RIPA buffer with protease inhibitor (Thermo Scientific) from Boston BioProducts was used to harvest protein lysates from the transfected MBA-MD231 cells. The cell lysate was centrifuged at 13,000*g* for 5 min followed by the removal of precipitates. The supernatant was subjected to BCA protein assay to determine the protein concentration. For western blot analysis, 30 μg of protein was used.

The transfected cells were harvested, and the RNAs were purified at day 4 via the TRIzol™-chloroform extraction. For one well in a six-well plate, 500 μL of TRIzol™ was added into the well to lyse cells and stabilize RNAs, followed by addition of chloroform. The upper layer of the phenol-chloroform was used for column-based RNA purification (RNeasy from Qiagen). One microgram of total RNA was used for cDNA synthesis by iScript™ cDNA synthesis kit from Bio-Rad, which contains both oligo dT and random hexamers. The levels of circMYC were detected by the following divergent primers which detect only circularized MYC transcript using RT-qPCR: F 5′-CATCAGCACAACTACGCAGC-3′ and R 5′-TCCAGCAGAAGGTGATCCAG-3′. 18s rRNA was used as internal control: F 5′-GTAACCCGTTGAACCCCATT-3′ and R 5′-CCATCCAATCGGTAGTAGCG-3′. For the cell proliferation assay, WST-1 from Sigma Aldrich was used from days 1, 2, 3, and 4.

### Associations between drug response and expression of circRNAs

We analyzed matched cell lines with both circRNA profiling and drug response data. For each individual circRNA, we first classified the cell lines into two groups based on the expression pattern of the circRNAs, based on if it exceeded the median value of backsplicing reads of all cell lines. We applied the Wilcoxon rank-sum test to identify individual drugs associated with the circRNA expression profile and quantify the difference in the mean AUC between the two groups. To correct the outcome of multiple tests, we used the Benjamini-Hochberg corrections. We defined a significant association between drug sensitivity and the circRNA profile with FDR < 0.05.

## Results

### Expression landscape of circRNAs across ~ 1000 cancer cell lines

**Fig. 1:**
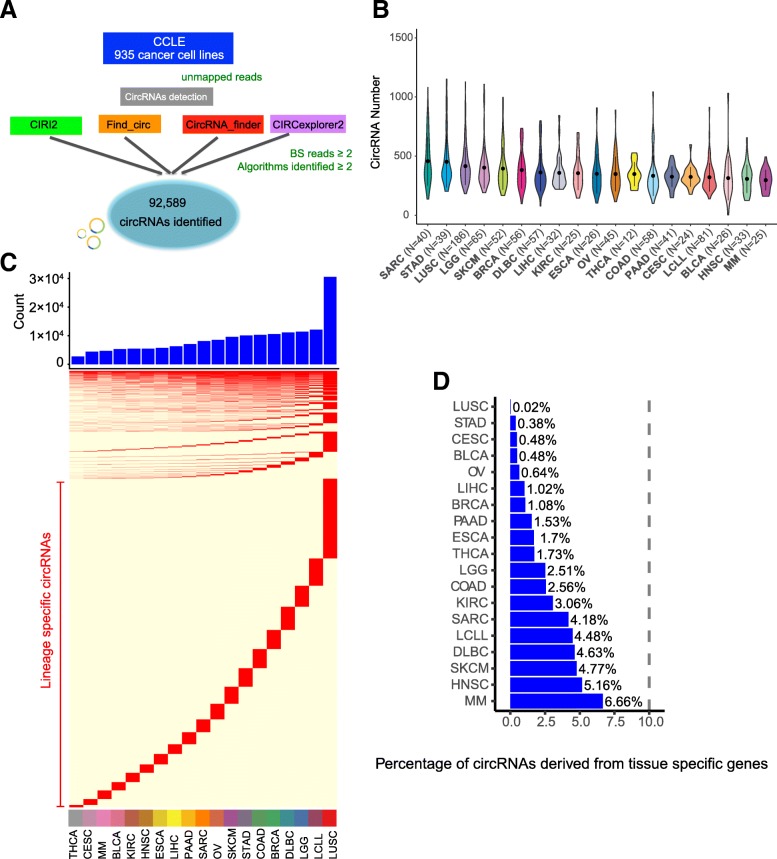
Global expression landscape of circRNAs across cancer cell lines. **a** Combination of four computational pipelines to identify circRNAs in Cancer Cell Line Encyclopedia (CCLE) RNA-seq data. **b** Violin plot shows number of circRNAs identified in cancer lineages with at least 10 cancer cell lines. **c** Tissue-specific circRNA profile across cancer lineages. The blue bar represents the total circRNA number for each cancer lineage. **d** Small percentage of lineage-specific circRNAs are derived from lineage-specific mRNAs

To systematically investigate the global circRNA landscape in cancer cell lines, we combined four well-established, user-friendly computational algorithms [13, 32], CIRI2 [33], Find_circ [3], CircExplorer2 [34], and CircRNA_finder [35], to identify backsplice-spanning reads from 935 cancer cell lines across 22 cancer lineages. The number of cell lines in each cancer lineage and names of the cancer lineages are listed in Additional file 1: Table S1. Since different algorithms identified varied numbers of circRNAs [42] (Additional file 1: Figure S1A), we required the circRNAs to be supported by at least two methods with backsplicing reads ≥ 2, and we identified in total 92,589 circRNAs (Fig. 1a and Additional file 1: Figure S1B). In each cancer cell line, we identified an average of 374 circRNAs, ranging from 78 in WSU-DLCL, a B cell lymphoma cell line (DLBC), to 1341 in JHUEM-1, an endometrial adenocarcinoma cell line (UCEC, Fig. 1b). For example, MCF7, the most studied human breast cancer cell line (BRCA), contained 379 circRNAs, and MDA-MB-231, a triple-negative breast cancer cell line, contained 226 circRNAs, while A549, the cancer cell line most widely used for non-small cell lung cancer research, contained 893 circRNAs.

We further examined the expression pattern of circRNAs across cancer cell lineages. We identified 1108 circRNAs (1.20%) ubiquitously expressed in ≥ 15 cancer lineages. For example, circDNMT1 derived from DNMT1, which is a DNA methyltransferase with essential roles in mammalian development [43], was expressed in all the lineages. CircNotch2 derived from Notch2, an important therapeutic target in cancer treatment [44], was also expressed in all the lineages. Interestingly, 69,656 (75.2%) of the circRNAs were expressed exclusively in one cancer lineage, indicating that circRNAs are more likely to be lineage-specific (Fig. 1c). The lineage-specific circRNAs ranged from 627 (22.9%) in thyroid carcinoma to 16,533 (54.1%) in lung squamous cell carcinoma. Notably, these lineage-specific circRNAs cover a number of cancer-related genes. For example, circPIK3CB is specifically identified in the colon adenocarcinoma cancer lineage. PIK3CB is involved in the phosphoinositide 3-kinase (PI3K) pathway, which is frequently genetically altered in human colon and rectal cancer and has been identified as a potential therapeutic target [45]. Another attractive therapeutic target is ROS1, a proto-oncogene receptor tyrosine kinase [46]; we found circROS1 to be specifically identified in the sarcoma. This lineage-specific circRNA pattern can indicate important clues about its physiological function.

To examine whether lineage-specific circRNAs are introduced by their linear mRNA genes, we obtained lineage-specific mRNA genes as previously described [36]. We observed that only 0.02 to 6.66% of lineage-specific circRNAs are derived from the lineage-specific mRNA genes (Fig. 1d), suggesting that lineage-specific circRNAs are independent from the expression of their parental mRNA gene. We also examined the correlation between the average number of lineage-specific circRNAs and the average total number of mappable reads across cancer lineages and observed no significant correlation (*Rs* = 0.08 and *p* = 0.70; Additional file 1: Figure S1C), indicating that the coverage is not a potential bias in identifying lineage-specific circRNAs.

### Identification of key genes and biological processes for the biogenesis of circRNAs

**Fig. 2:**
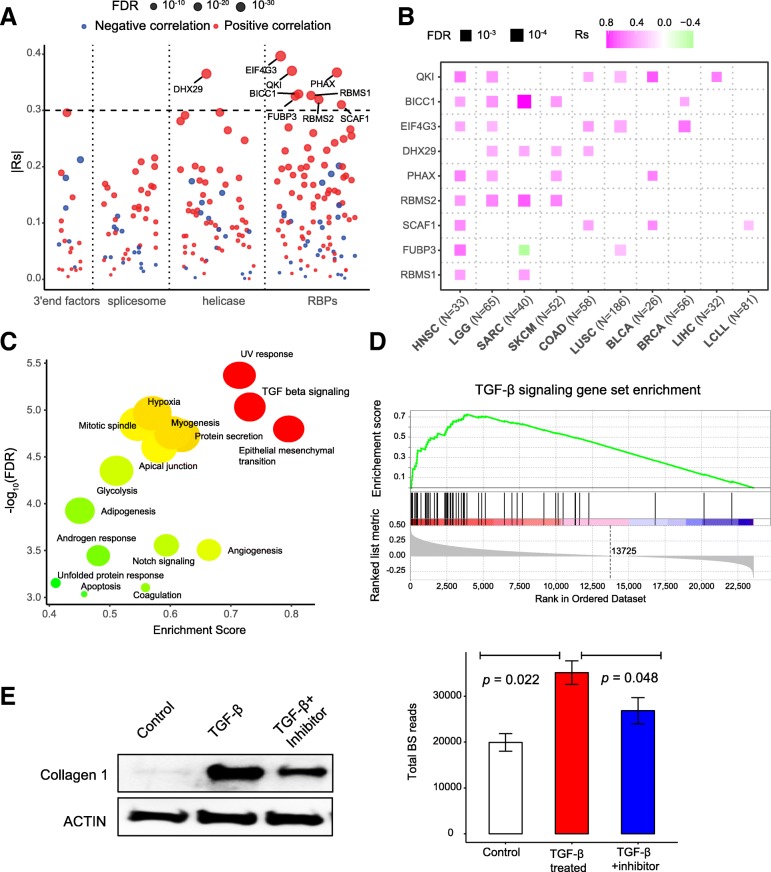
Regulators in biogenesis of circRNAs. **a** Correlation between each potential factor and normalized total circRNA backsplicing reads among all cancer cell lines. **b** Correlation between top 9 significant genes and normalized total circRNA backsplicing reads across multiple cancer lineages. The color represents the Spearman coefficient value; size represents the false discovery rate. **c** Gene set enrichment analysis (GSEA) of all 50 hallmark gene sets based on circRNA expression profile. **d** GSEA of TGF-β gene set based on circRNA expression profile across 935 cancer cell lines. **e** Cells were treated with TGF-β or TGF-β + SIS3. The COL1A1 and ACTIN protein levels were examined by western blot (left panel); bar plot represents total number of backsplicing reads for circRNAs within each sample (right panel)

The biogenesis of circRNAs is regulated by multiple factors, including *cis*-regulatory elements and *trans*-acting proteins [4]; however, the underlying mechanisms are still not fully understood. Here, we take advantage of this large-scale circRNA profile to identify key regulators in circRNA biogenesis. We focused on several groups of genes that have been reported as being involved in circRNA biogenesis, including spliceosome factors (*n* = 51) [47], 3′ end processing factors (*n* = 22) [47], RNA helicases (*n* = 71) [8], and a subset of RNA-binding proteins with potential effects on the biogenesis of circRNAs (RBPs, *n* = 104) [48]. Nine genes were significantly correlated with total backsplicing reads across all cancer cell lines (|*Rs|* > 0.3, FDR < 0.05), suggesting their significant roles in the biogenesis of circRNAs (Fig. 2a). We also identified RBPs negatively correlated with total backsplicing reads, which is consistent with a previous study [7]. Most of these genes showed strong positive correlations across multiple cancer lineages (Fig. 2b). For example, QKI showed positive correlation with the biogenesis of circRNAs in six cancer lineages (Fig. 2b). This is consistent with the previous discovery that upregulation of QKI can induce de novo circRNA biogenesis in breast cancer cells [6].

To further explore other potential biological processes involved in circRNA biogenesis, we performed gene set enrichment analysis (GSEA) using 50 “hallmark” gene sets from the Molecular Signature Database that represent major biological processes [37]. All protein-coding genes (*n* = 23,714) available in the gene expression matrix of CCLE are used as background for GSEA. We identified 16 significant related biological processes that may contribute to the biogenesis of circRNAs, such as EMT, which is consistent with a previous report [6] (Fig. 2c). Indeed, the number of circRNA reads is highly correlated with EMT score based on canonical EMT markers [31] (Spearman correlation *Rs* = 0.37, *p* < 2.2 × 10^− 16^; Additional file 1: Figure S2A). The number of circRNA reads is also significantly higher in cancer cell lines with higher EMT score (Wilcoxon rank-sum test *p* = 5.5 × 10^− 16^; Additional file 1: Figure S2B). Interestingly, we also found that the TGF-β pathway, an important signaling pathway in cancer development [49], is significantly enriched in cancer lineages with high numbers of backsplicing reads (Fig. 2d). We treated the A549 human lung carcinoma cells with TGF-β and confirmed the activation of TGF-β signaling by the induction of Collagen 1 (Fig. 2e, left panel). Indeed, the total number of backsplicing reads increased significantly with TGF-β treatment (Student’s *t* test, *p* = 0.022; Fig. 2e, right panel). Treatment with SIS3, a TGF-β signaling inhibitor [50], markedly reduced the induction of COL1A1 and decreased the total number of backsplicing reads, suggesting that SIS3 reversed the TGF-β-mediated enhancement of circRNA formation (Student’s *t* test, *p* = 0.048; Fig. 2e, right panel). This is consistent with a previous study using TGF-β treatment to induce epithelial–mesenchymal transition (EMT), which increased the circRNA abundance (Additional file 1: Figure S2C) [6]. Taken together, our data further confirmed that activation of the TGF-β signaling pathway could promote the biogenesis of circRNAs.

### Characterization of circRNAs generated from clinically actionable genes

**Fig. 3:**
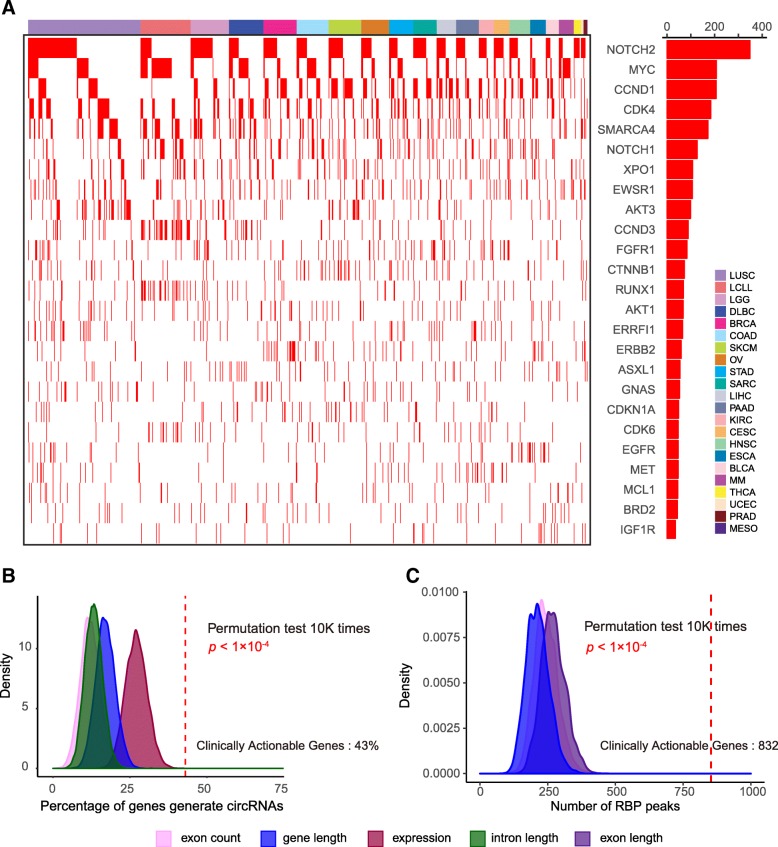
Characterization of circRNAs generated from clinically actionable genes. **a** circRNA expression pattern in clinically actionable genes across cancer lineages. Red bars on the right panel represent the total number of cancer cell lines that identified each circRNA. **b** Permutation test of enrichment of circRNAs compared with randomly selected background gene list. Randomly selected background genes were sampled from genes with similar length, intron length, number of exons, or gene expression level. Vertical red lines indicate the percentage of clinically actionable genes that generated circRNAs. **c** Permutation test of number of RBP binding peaks on clinically actionable genes with randomly selected background gene list. Randomly selected background genes were sampled from genes with similar length, number of exons, or exon length. Vertical red lines indicate the median number of RBP binding peaks of clinically actionable genes

Clinically actionable genes in cancer research are genes and/or associated genes that have been approved by the US Food and Drug Administration as drug targets for cancer therapy [46]. We identified 59 (43.7%) clinically actionable genes that could form circRNAs in at least one cancer cell line (Fig. 3a). For example, Notch2, the key gene in the notch signaling pathway [51], has had circRNAs identified in 349 (37.3%) cancer cell lines. MYC, an oncogene that contributes to the progression of many human cancers, has had circRNAs identified in 208 (22.2%) cancer cell lines. Interestingly, clinically actionable genes are more likely to generate circRNAs (43.7% vs. 19.4%, Pearson’s chi-squared test *p* < 2.2 × 10^− 16^; Additional file 1: Figure S3A), and this enrichment is not introduced by gene length (permutation test, *p* < 1 × 10^− 4^), intron length (*p* < 1 × 10^− 4^), number of exons (*p* < 1 × 10^− 4^), or gene expression level (*p* < 1 × 10^− 4^, Fig. 3b). To further understand the enrichment of circRNAs in clinically actionable genes, we examined a batch of experimentally validated RNA-binding protein (RBP) binding peaks [40] because RBP could potentially affect the biogenesis of circRNA [48]. We found clinically actionable genes have more RBP binding sites (median 853 vs. 210, Wilcoxon rank-sum test *p* < 2.2 × 10^− 16^; Additional file 1: Figure S3B), and this is not introduced by confounding factors of gene length (permutation test, *p* < 1 × 10^− 4^), exon length (*p* < 1 × 10^− 4^), or number of exons (*p* < 1 × 10^− 4^, Fig. 3c). This result suggested that the enrichment of circRNAs in clinically actionable genes is possibly due to the enrichment of RBP binding sites.

### Functional effects and drug response of circMYC in cancer

**Fig. 4:**
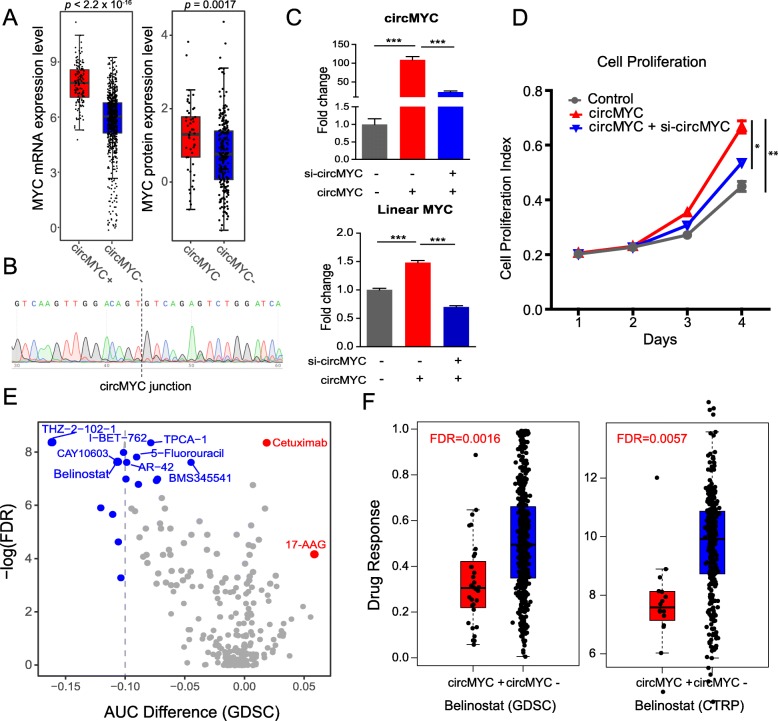
Functional effects and drug response of circMYC in cancer. **a** Overexpression of MYC mRNA level (left panel) and protein level (right panel) in cancer cell lines with expression of circMYC. **b** Sanger sequencing confirmed that PCR products spanned the circular junction of predicted circMYC. The black dash line indicates the circular junction site. **c** RT-qPCR for the overexpression and siRNA knockdown of circMYC in MDA-MB-231 cell for circMYC (upper panel) and MYC mRNA level (lower panel). **d** Cell proliferation assay in MDA-MB-231 cells transfected with circMYC and with specific circMYC knockdown. **e** The association between circMYC and response of diverse drugs (AUC) from GDSC dataset. **f** Comparison of Belinostat response between circMYC-positive and circMYC-negative cells from GDSC and CTRP dataset (FDR calculated from Wilcoxon rank-sum test with multiple adjustment). *: *p* < 0.05; **: *p* < 0.01; ***: *p* < 0.001

To further explore the functional effects of circRNAs in human cancer, we focused on circMYC (chr8:128752711-128752871), which is derived from one of the most important cancer genes. Interestingly, circMYC is significantly more abundant than its paralogous genes (22.2% vs. circMYCL 2.4% and circMYCN 0.2%, Pearson’s chi-squared test *p* < 2.2 × 10^− 16^, Additional file 1: Figure S4A). We found that both the MYC mRNA level (Student’s *t* test *p* < 2.2 × 10^− 16^) and protein level (Student’s *t* test *p* = 0.005) are significantly higher in the group with circMYC expression (Fig. 4a). To further experimentally verify the effect of circMYC on MYC protein expression, we overexpressed circMYC by transferring a synthetic circMYC expression vector into MDA-MB-231, a human breast cancer cell line. We observed the signal for circMYC with or without treatment of RNase R (Additional file 1: Figure S4B), and the Sanger sequencing further confirmed that the PCR products spanned the circular junction of predict circMYC (Fig. 4b), suggesting that our circMYC is a bona fide circRNA. We then confirmed the overexpression of circMYC by using divergent primers that specifically amplified the backspliced sites of circMYC (Fig. 4c, upper panel). We observed the overexpression of MYC mRNA (Fig. 4c, lower panel) and protein (Additional file 1: Figure S4C) upon overexpression of circMYC. Furthermore, the overexpression of circMYC led to a significant increase in cell proliferation, compared to cells transfected with a control vector (Fig. 4d). SiRNA knockdown of circRNA led to a decrease in cell proliferation, confirmed the specific effects of overexpressed circMYC (Fig. 4d). Taken together, these results suggest that circMYC play significant roles in cell proliferation, potentially through mediating MYC gene.

We further explored the effects of circMYC on drug response from Genomics of Drug Sensitivity in Cancer (GDSC) with 265 drugs for 644 human cancer cell lines [27], and Cancer Therapeutics Response Portal (CTRP) with 481 drugs for 860 cancer cell lines [28]. CircMYC are significantly associated with drug response of multiple drugs, including Belinostat, THZ−2−102−1, Cetuximab among the top ones in GDSC (Fig. 4e). Among these significantly associated drugs, Belinostat, a HDAC inhibitor, showed consistent sensitive with circMYC in both datasets of GDSC (Wilcoxon rank-sum test with multiple adjustment, FDR = 0.0016, Fig. 4f) and CTRP (FDR = 0.0057, Fig. 4f). We also observed that circMYC expression was consistently sensitive towards treatment with Vorinostat, another HDAC inhibitor in both datasets of CTRP (FDR = 0.0032) and GDSC (FDR = 0.00048, Additional file 1: Figure S4D). However, due to the nature of drug response data with large spread [19, 52], further in vitro and in vivo investigation is necessary to select appropriate treatment options. Our results highlighted the possibility that circMYC generated from MYC may affect drug response in ways that are beyond known mechanisms, including genomic variation [23] and transcriptomic variation [22].

### Therapeutic liability of circRNAs

**Fig. 5:**
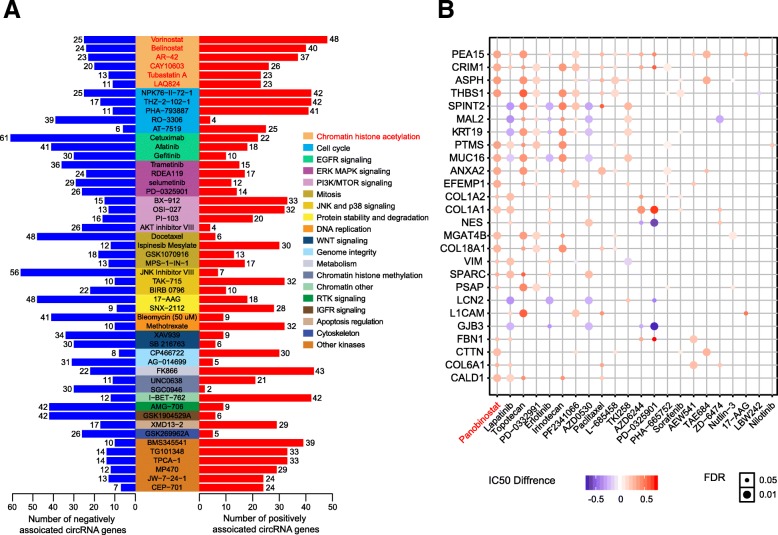
Therapeutic liability of circRNAs in GDSC and CCLE. **a** Significantly associated circRNA–drug pairs in Genomics of Drug Sensitivity in Cancer (GDSC) dataset. Blue bar denotes negative association; red bar denotes positive association. **b** Association of circRNA profiles with drug sensitivity in Cancer Cell Lines Encyclopedia (CCLE) dataset. The circle color represents the mean AUC difference between circRNA-positive and circRNA-negative groups; circle size represents false discovery rate

We further performed systematic analysis to examine the effects of circRNAs on drug response from the GDSC drug dataset. We identified 4564 circRNA–drug pairs with statistically significant association (false discovery rate [FDR] < 0.05). For example, cellular sensitivity to the drug Vorinostat, a HDAC inhibitor, is positively associated with 48 circRNAs and negatively associated with 25 circRNAs (Fig. 5a). We focused on drugs highly associated with circRNAs (*n* > 30) and examined the pharmaceutical targets of these drugs. Interestingly, we found that the top drug category is drugs that target chromatin histone acetylation. For example, 6 of 11 HDAC inhibitors are associated with circRNAs (Fig. 5a). To further validate this finding, we examined the effects of circRNAs on drug sensitivity using another independent dataset from CCLE, which included 24 drugs on 468 cancer cell lines [19]. We identified 343 significantly associated circRNA–drug pairs (FDR < 0.05). The top drug associated with circRNAs is Panobinostat, a HDAC inhibitor (Fig. 5b). The high correlations between circRNAs and HDAC inhibitors are also identified in drug response database of CTRP (Additional file 1: Figure S5). For instance, Vorinostat, a HDAC inhibitor, is positively associated with 100 circRNAs and negatively associated with 48 circRNAs in CTRP. This consistency highlighted the close link between chromatin histone acetylation inhibition drugs and circRNA expression.

### A user-friendly data portal for integrative analysis of circRNAs with multi-omics data in cancer cell lines

**Fig. 6:**
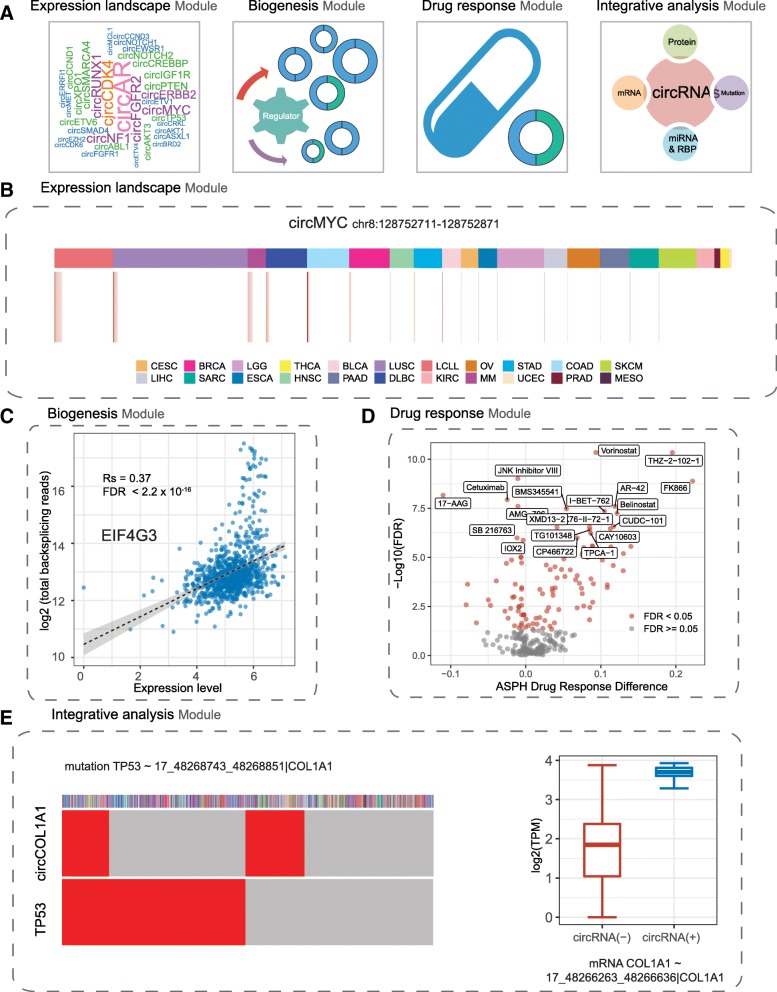
Overview of CircRiC data portal. **a** Graphic scheme of CircRic data portal. **b**–**e** Four functional modules and graphic examples in CircRiC: expression landscape of circRNA across cancer cell lines (**b**), biogenesis of circRNA (**c**), drug response associations between drug response and circRNAs (**d**), integrative analysis of circRNA with multi-omics features, including mutations, mRNA expression, or protein expression (**e**)

To facilitate the exploration of the function of circRNAs across ~ 1000 cancer cell lines by the wider research community, we developed a user-friendly data portal, CircRNAs in cancer cell lines (CircRiC, https://hanlab.uth.edu/cRic). In this portal, we provide circRNA expression data and the association between circRNA expression and multi-omic data, including mRNA expression, proteomic, mutation, and drug sensitivity data. CircRiC enables users to examine the features of circRNAs in cancer cell lines in a flexible and interactive way. CircRiC provides four interactive modules: expression landscape, biogenesis, drug response, and integrative analysis (Fig. 6a). The expression landscape module provides the circRNA expression profile across all cancer cell lines (Fig. 6b, Additional file 1: Figure S6A). The biogenesis module shows the genome-wide correlation of the expression of individual genes with the total backsplicing reads (Fig. 6c, Additional file 1: Figure S6B), which indicates the potential role of genes in circRNA biogenesis. The drug response module provides the association between circRNAs and drug sensitivity using GDSC, CCLE, and CTRP drug response datasets (Fig. 6d, Additional file 1: Figure S6C). In the integrative analysis model, we systematically analyze the associations between circRNAs and mRNA, proteins, or mutations. We identified 2649 significant circRNA–protein associations, 9604 circRNA–mRNA associations, and 117,258 circRNA–mutation associations (Fig. 6e). With the module, users can easily examine whether one circRNA is associated with various types of molecular data. For example, circKRT19 expression derived from KRT19, a notch signaling regulator in breast cancer [53], is significantly associated with higher ERBB3 expression in mRNA (Wilcoxon rank-sum test, FDR < 2.2 × 10^− 16^) and protein levels (Wilcoxon rank-sum test, FDR = 7.91 × 10^− 11^) (Additional file 1: Figure S6D). The expression of circKRT19 is negatively associated with ERBB3 mutation (FDR = 6.66 × 10^− 12^) (Additional file 1: Figure S6D), suggesting latent mutual exclusivity. ERBB3 is a member of the receptor tyrosine kinase family and is involved in the development of numerous types of human cancer [54]. This result has implicated circKRT19 in the regulation of ERBB3 function and in cancer development. In addition, we provide predicted miRNA- and RNA-binding protein binding sites for each circRNA. In summary, CircRiC provides multiple layers of circRNA-related data for browsing, analyzing, visualizing, and downloading. This valuable resource will significantly contribute to research on circRNAs.

## Discussion

CircRNAs have been found to be ubiquitous in human cancers [12, 55]. Using large cancer cell-line datasets, we systematically characterized circRNA profiles across multiple cancer lineages. Different algorithms adopt distinct strategies to detect backsplicing events (where circular RNAs are produced) from RNA sequencing reads [56, 57]. In particular, CIRI2 examines the paired chiastic clipping signals from the mapping information, while Find_circ predicts the backsplicing events from the first and last 20-bp anchors of unmapped reads. In addition, CircExplorer2 and CircRNA_finder both employed split-alignment aligners to parse backsplicing events. These algorithms have been comprehensively evaluated in previous literatures [32, 33, 56]. As expected, these algorithms yielded divergent circRNA predictions. Therefore, we combined the outputs of these four tools to reduce false positive predictions of circRNAs. We found that the majority of circRNAs can only be identified in one cancer lineage, and the lineage specificity is independent from the parental mRNA gene expression. This result revealed that circRNAs are specifically generated in different cancer lineages, which may further contribute to tumor heterogeneity.

We further explored the key genes involved in circRNA biogenesis. We identified 9 RBPs that are significantly associated with global circRNA biogenesis, which is consistent with a previous study, that RBPs are key regulators of tissue-specific pattern in circRNAs [7]. Other than known factor QKI, we identified several novel RBPs that could potentially contribute to promote circRNA biogenesis. Besides individual factors, we also confirmed TGF-β signaling to be involved in circRNA formation as previously described [6].

Furthermore, we observed that clinically actionable genes are more likely to generate circRNAs. This is possibly due to the enrichment of RBP binding sites, which is independent from gene length, intron length, number of exons, or gene expression level. Further investigation is necessary to understand the underlying mechanism and clinical utility of the enrichment of circRNAs in clinically actionable genes. Among those clinically actionable genes, we demonstrated the functional roles of circMYC to increase cell proliferation through mediating MYC gene, a key oncogene across multiple cancer types [58]. Our results suggest a new regulatory layer of the MYC gene through circRNAs and highlight the significant roles of circMYC in tumorigenesis.

CircRNAs may also affect the efficacy of drug treatments. In this study, we validated the drug response through multiple large-scale pharmacologic data. In particular, we observed that the circMYC expression is correlated with the drug response of Belinostat and Vorinostat, two HDAC inhibitors, in both GDSC and CTRP datasets. We observed strong association between the response to drugs that target chromatin histone acetylation and the expression of circRNAs. Previous studies showed a significant association between circRNA abundance and chromatin-bound [5], which might explain our observations. Further investigation is necessary to understand the connections between circRNAs and chromatin epigenetic changes.

CCLE generated RNA-seq data based on polyA-selection library, which may not be the ideal library preparation strategy for circRNA. However, CCLE RNA-seq data represents the only resource in such a large magnitude, and we did detect a considerate number of circRNAs across cell lines (median number 339, and maximum number 1341). Furthermore, the recent release of next-generation CCLE facilitates the development of novel therapeutic strategies [19]. With the appropriate RNA library, sequence depth, and read length, it is possible to identify more circRNAs [56], but it is appropriate to compare the same circRNAs across cell lines instead of comparing different circRNAs.

To facilitate circRNA research in the community, we constructed an interactive and user-friendly data portal, CircRiC, which provides comprehensive analysis of circRNAs with multidimensional data across ~ 1000 cancer cell lines. Our data resource is a complement to MiOncoCirc, which covered circRNA expression from more than 2000 tumor samples and a few cancer cell lines (*n* = 28) [17]. We expect that CircRic will help researchers to better select the appropriate cancer cell lines to understand the role of circRNAs in cancers.

## Conclusions

In conclusion, we systematically characterized circRNAs in ~ 1000 cancer cell lines and demonstrated the lineage specificity of circRNAs across multiple human cancer lineages. We identified potential factors involved in circRNA biogenesis and confirmed that TGF-β signaling promotes circRNA formation. We observed that clinically actionable genes are likely to generate more circRNAs, potentially due to the enrichment of RBP binding sites. We further characterized the functions of circMYC in promoting cell proliferation and revealed the potential link between circRNA expression and pharmaceutical profiling. Finally, we developed a user-friendly data portal CircRic that integrates our data to benefit the research community.

## Additional file

Additional file 1: **Figure S1.** Expression landscape of circRNAs across cancer cell lines. **Figure S2.** Potential regulation of EMT on biogenesis of circRNAs. **Figure S3.** Enrichment of circRNAs in clinically actionable genes. **Figure S4.** Characterization of functional effects and drug response of circMYC in cancer. **Figure S5.** Therapeutic liability of circRNAs in CTRP. **Figure S6.** Examples of modules in CircRiC. **Table S1**. Summary of circRNAs identified across different cancer cell lines. (PDF 7931 kb)

## Data Availability

The RNA-seq data for TGF-β treatment have been submitted to the NCBI Gene Expression Omnibus (GSE119145). All other data is available at CircRic data portal (https://hanlab.uth.edu/cRic).
