# Supplementary material for: Comprehensive characterization of circular RNAs in ~ 1000 human cancer cell lines
Source: Genome Med. 2019 Aug 26;11:55. doi: 10.1186/s13073-019-0663-5 (PMC6709551; doi:10.1186/s13073-019-0663-5)
Supplement: Supplementary file 1 — Figure S1. Expression landscape of circRNAs across cancer cell lines. Figure S2. Potential regulation of EMT on biogenesis of circRNAs. Figure S3. Enrichment of circRNAs in clinically actionable genes. Figure S4. Characterization of functional effects and drug response of circMYC in cancer. Figure S5. Therapeutic liability of circRNAs in CTRP. Figure S6. Examples of modules in CircRiC. Table S1. Summary of circRNAs identified across different cancer cell lines. (PDF 7931 kb) [file 13073_2019_663_MOESM1_ESM.pdf]

Figure S1

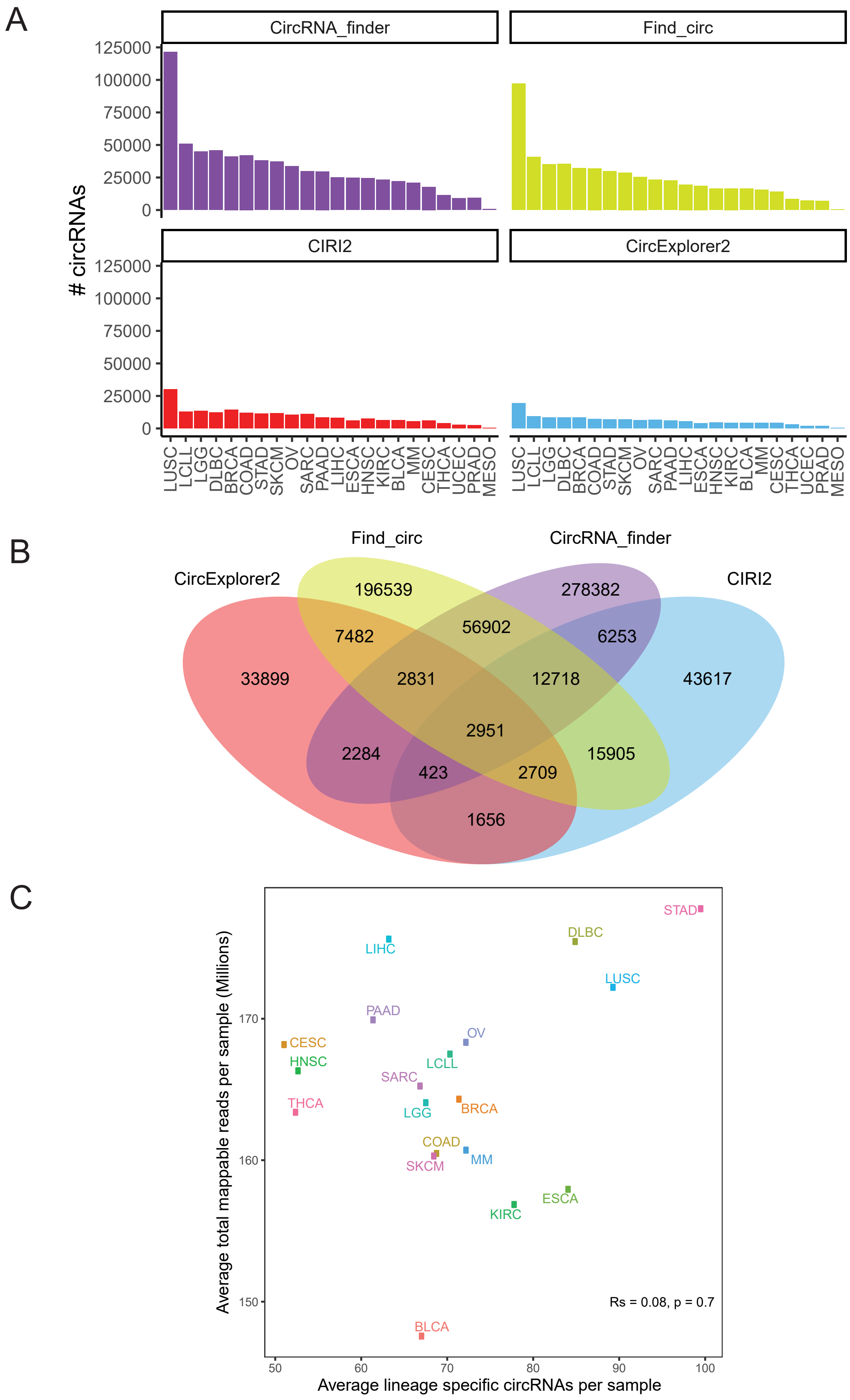

Figure S1. Expression landscape of circRNAs across cancer cell lines.
(A) Numbers of circRNAs detected by four circRNA identification algorithms across cancer cell lines (CircRNA_finder, Find_circ, CIRI2, CircExplorer2). (B) Venn diagram depicting the overlap among the four algorithms. (C) No significant correlation (Rs = 0.08, p = 0.7) between average cancer lineage specific circRNA and average mappable reads across cancer lineages.

Figure S2

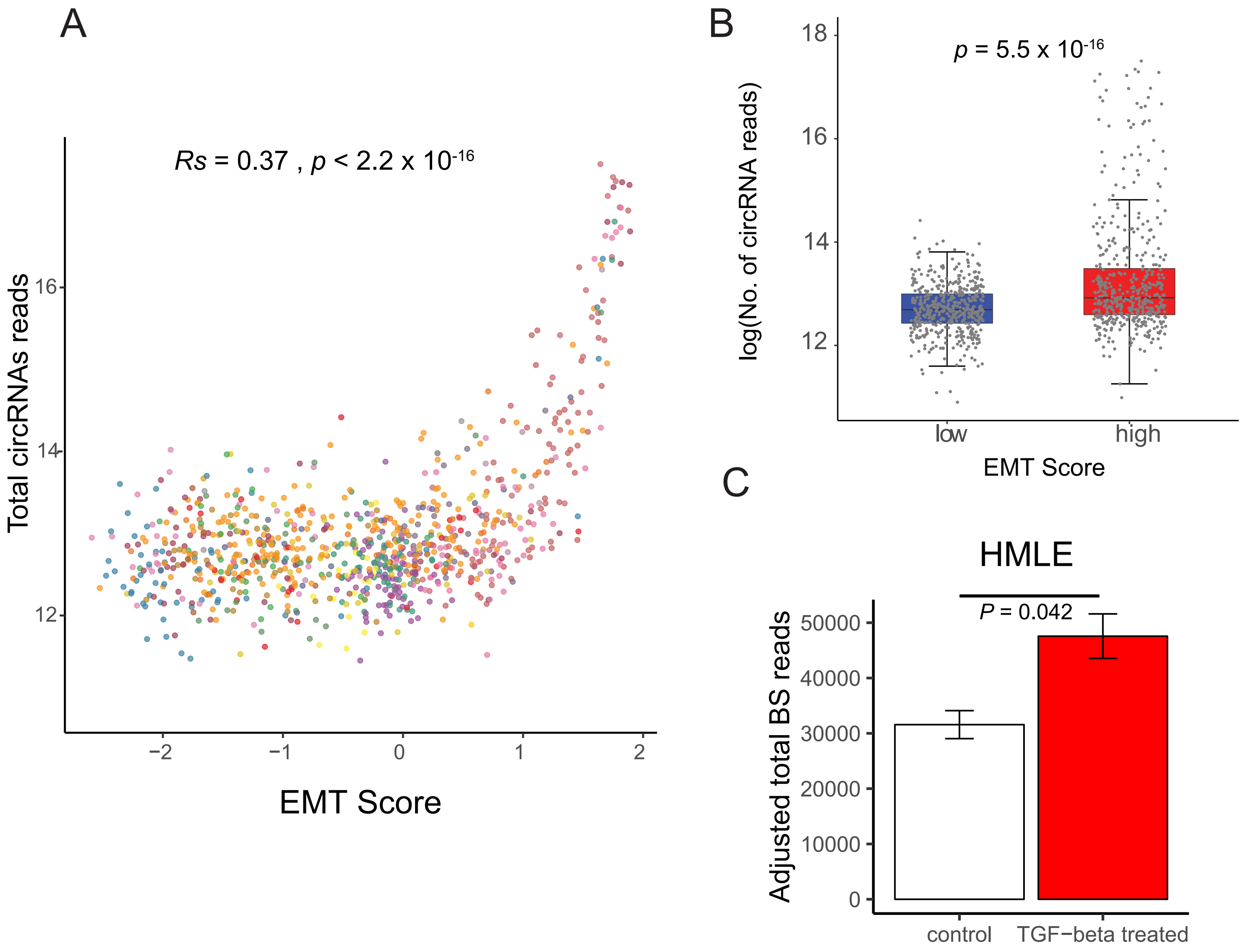

Figure S2. Potential regulation of EMT on biogenesis of circRNAs. (A) Significant correlation between EMT score and total circRNA backsplicing reads across cancer cell lines. (B) Increased number of circRNA backsplicing reads detected from high EMT Score cell lines compared to low EMT Score cell lines. (C) Increased backsplicing reads upon TGF-β treatment in human HMLE cells.

Figure S3

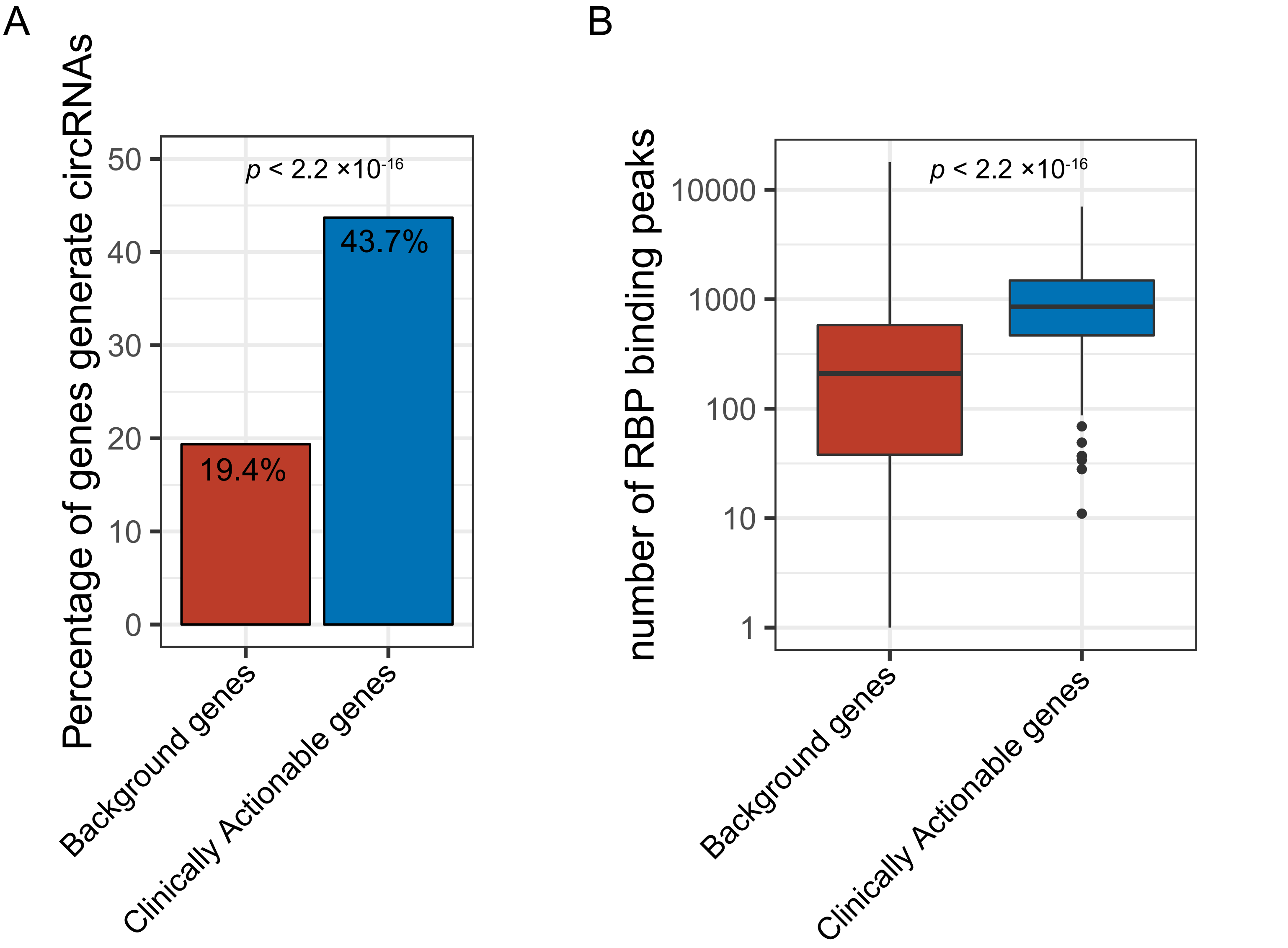

Figure S3. Enrichment of circRNAs in clinically actionable genes.
(A) Enrichment of circRNAs from clinically actionable genes compared with that of background genes (Pearson's Chi-squared test, $p < 2.2\times10^{-16}$). (B) Significantly higher number of RBP binding peaks of clinically actionable genes compared to that of background genes (Wilcoxon rank-sum test, $p < 2.2 \times 10^{-16}$).

Figure S4

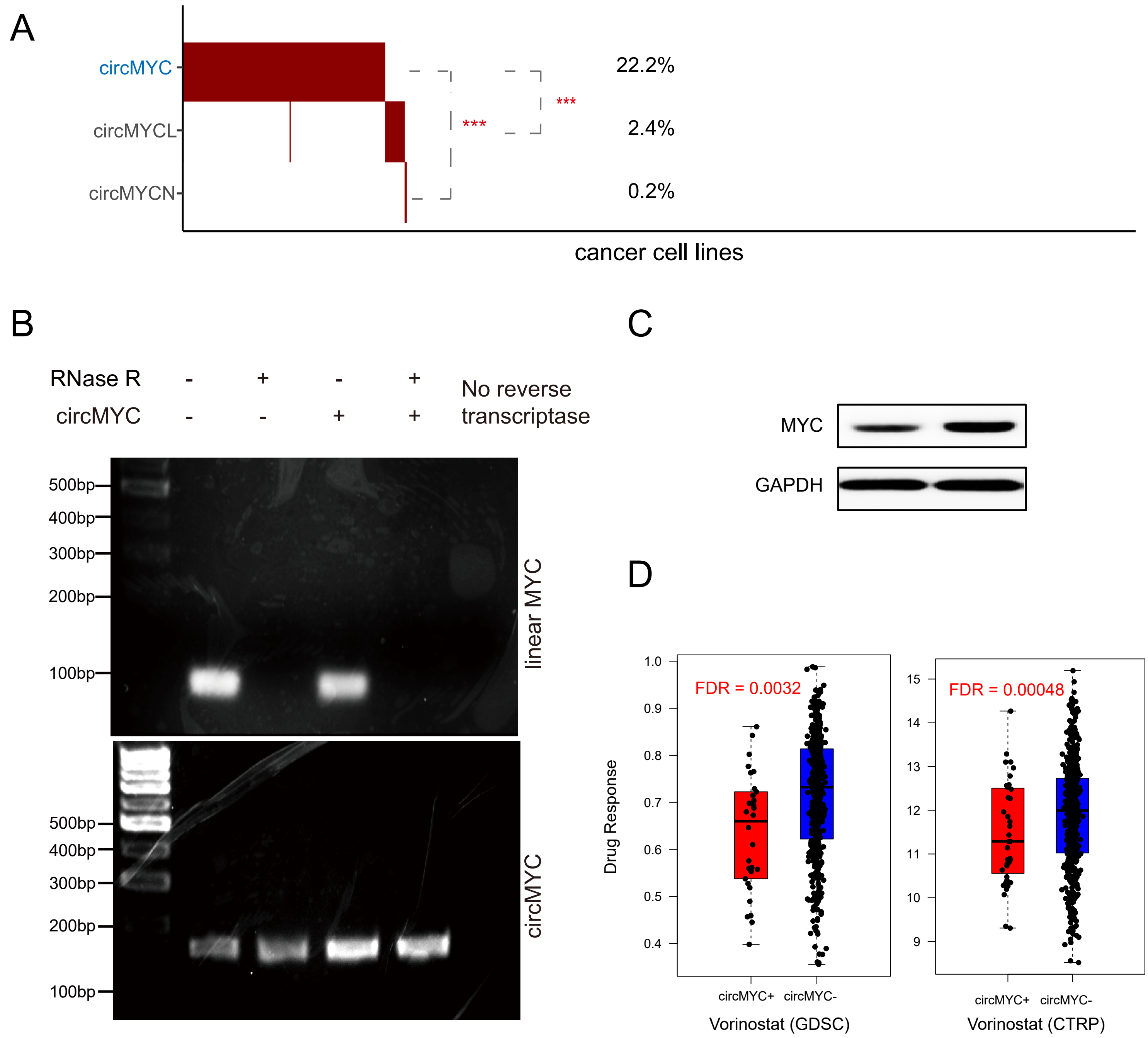

Figure S4. Characterization of functional effects and drug response of circMYC in cancer. (A) CircRNA expression of MYC, MYC human paralogous genes (MYCN and MYCL) across cancer cell lines. (B) PCR of linear MYC and circMYC with or without treatment of RNase R. (C) Overexpression of MYC protein level in MDA-MB-231 cells transfected with circMYC. (D) Comparison of Vorinostat response between circMYC positive and negative cells from GDSC and CTRP dataset (FDR calculated from Wilcox-on rank-sum test with multiple adjustment).
*** : $p < 0.001$

Figure S5

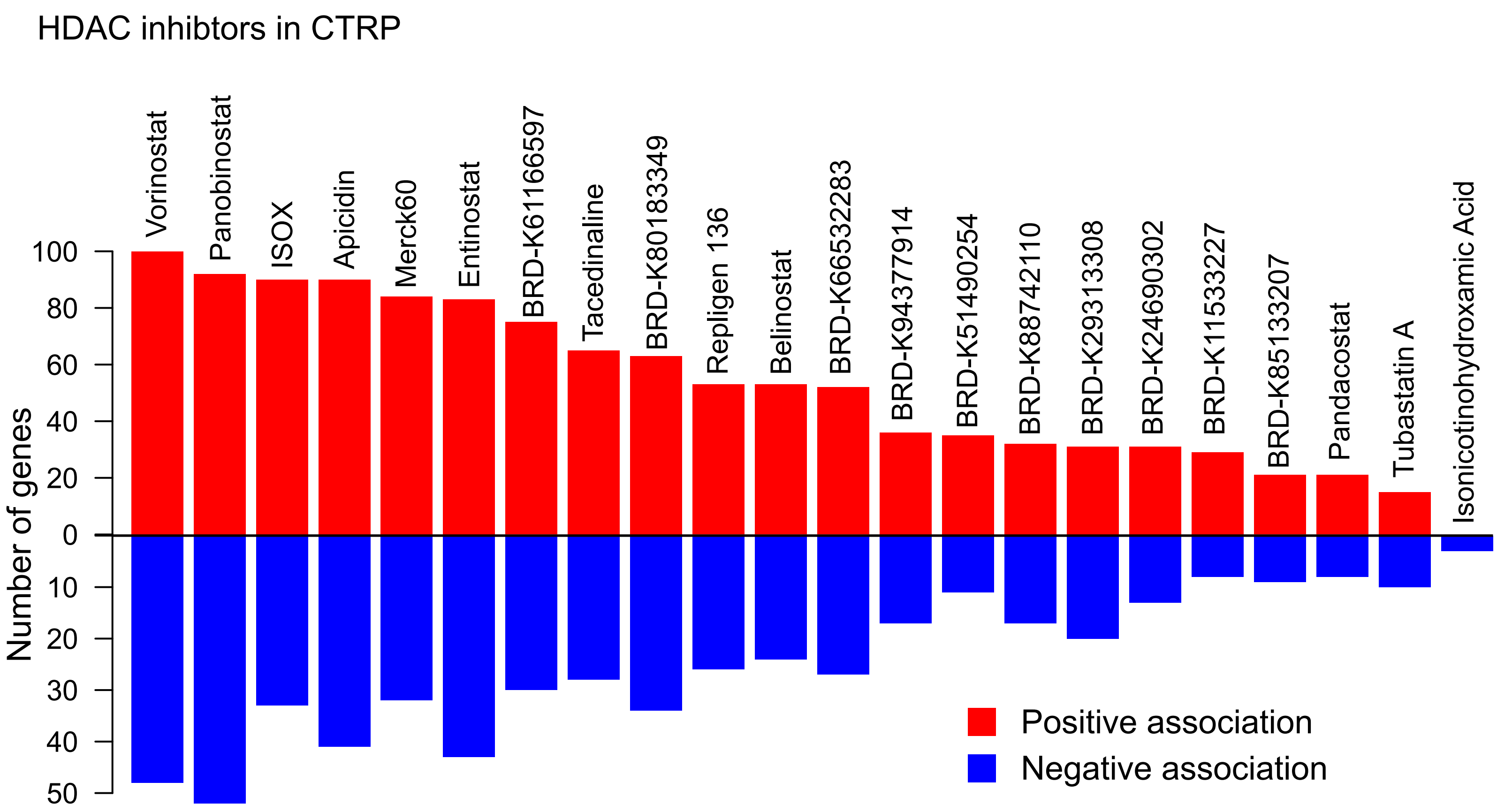

Figure S5. Therapeutic liability of circRNAs in CTRP.
The numbers of HDAC inhibitor drugs significantly associated with circRNAs in Cancer Therapeutic Response Portal (CTRP) dataset. Blue bar denotes negative association and red bar denotes positive association.

Figure S6

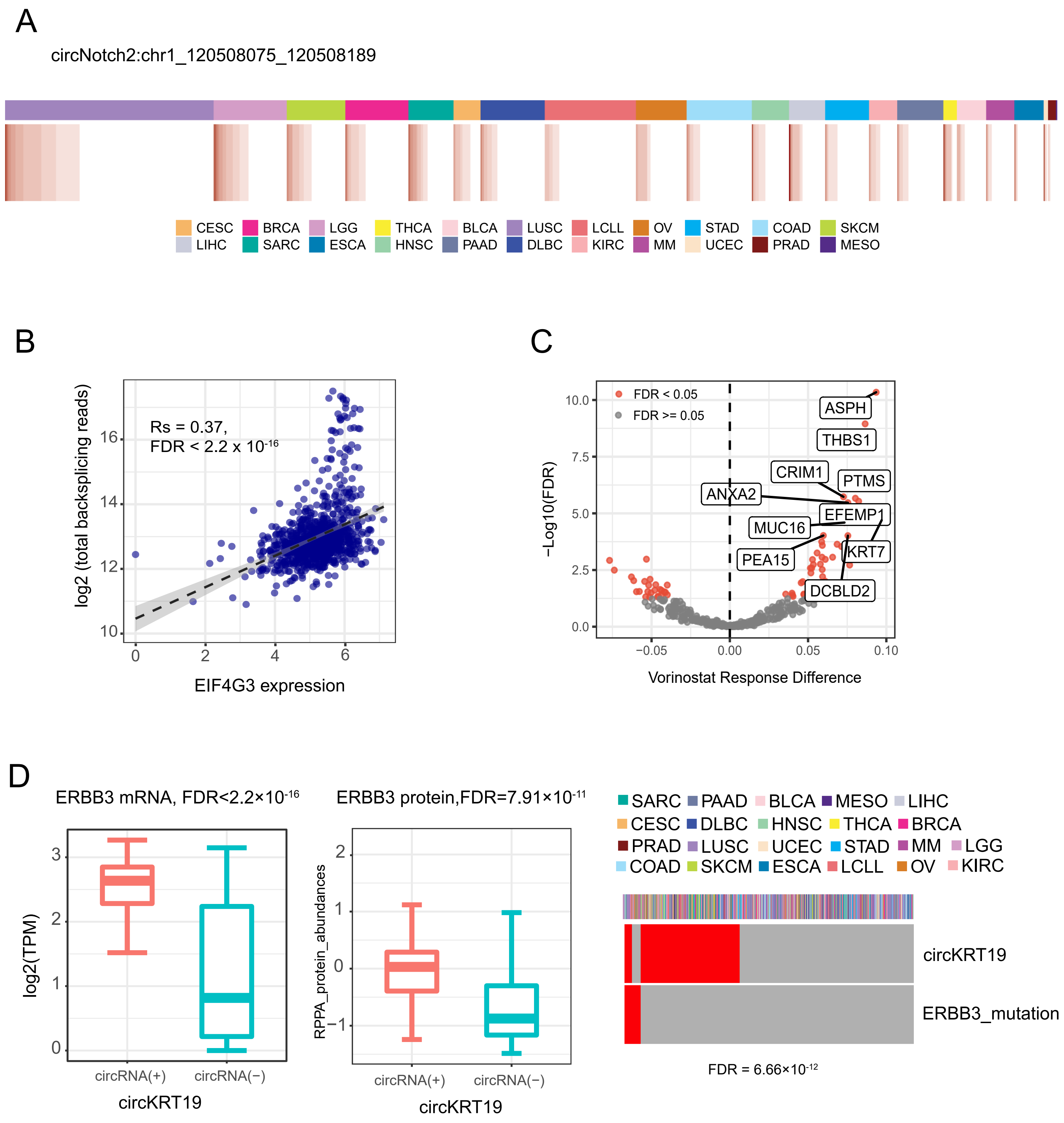

Figure S6. Examples of modules in CircRiC.

(A) Expression landscape of circMYC across different cancer cell lines. (B) Correlation of EIF4G3 gene expression with total backsplicing reads. (C) Association between circRNAs and sensitivity to vorinostat using GDSC drug–response dataset. (D) Associations between circKRT19 and ERBB3 mRNA, protein and mutation profile.

Table S1. Summary of circRNAs identified across different cancer cell lines

Cancer Lineages	No. of cell lines	No. of circRNA
**Lung squamous cell carcinoma [LUSC]**	186	77284
**Chronic lymphocytic leukemia [LCLL]**	81	26172
**Brain lower grade glioma [LGG]**	65	26038
**Colon adenocarcinoma [COAD]**	58	21450
**Diffuse large B-cell lymphoma [DLBC]**	57	20654
**Breast invasive carcinoma [BRCA]**	56	20525
**Skin cutaneous melanoma [SKCM]**	52	19366
**Ovarian serous cystadenocarcinoma [OV]**	45	18296
**Pancreatic adenocarcinoma [PAAD]**	41	17639
**Sarcoma [SARC]**	40	15715
**Stomach adenocarcinoma [STAD]**	39	13381
**Head and neck squamous cell carcinoma [HNSC]**	33	11476
**Liver hepatocellular carcinoma [LIHC]**	32	10157
**Bladder urothelial carcinoma [BLCA]**	26	9132
**Esophageal carcinoma [ESCA]**	26	8899
**Kidney renal clear cell carcinoma [KIRC]**	25	8177
**Multiple myeloma plasma cell leukemia [MM]**	25	7786
**Cervical squamous cell carcinoma and endocervical adenocarcinoma [CESC]**	24	7427
**Thyroid carcinoma [THCA]**	12	4186
**Prostate adenocarcinoma [PRAD]**	7	2704
**Uterine corpus endometrial carcinoma [UCEC]**	4	2684
**Mesothelioma [MESO]**	1	181
